# Type 1 Acute Aortic Dissection in the Early Period After COVID-19 Infection

**DOI:** 10.7759/cureus.13751

**Published:** 2021-03-07

**Authors:** Mesut Engin, Ufuk Aydın, Hacı Eskici, Yusuf Ata, Tamer Türk

**Affiliations:** 1 Cardiovascular Surgery, University of Health Sciences, Bursa Yuksek Ihtisas Training and Research Hospital, Bursa, TUR

**Keywords:** coronavirus/covid‐19, cardiovascular system, pandemic, inflammation, aortic dissection

## Abstract

The coronavirus disease 2019 (COVID-19) pandemic has resulted in over 105 million confirmed cases and over 2.3 million deaths globally as of February 3, 2021. Cardiovascular problems due to COVID-19 infection include acute coronary syndrome (due to coronary occlusion, thrombosis), myocardial damage without coronary artery disease, arrhythmias, heart failure, pericardial effusion, and thromboembolic events. A 62-year-old male patient was admitted to our emergency department with a complaint of chest pain radiating to his back. The patient had a history of hospitalization for seven days in the outpatient clinic and 10 days in the intensive care unit due to COVID-19 infection with severe lung involvement. In contrast-enhanced thoracoabdominal CT, a dissection line starting from the ascending aorta and progressing to the iliac bifurcation was observed. Ascending aorta and transverse arch replacement was performed with a 30-mm polytetrafluoroethylene tube graft. The patient was discharged home 15 days postoperatively.

## Introduction

Type 1 acute aortic dissection (AAD) is a serious clinical condition that requires urgent surgical intervention as it is associated with high mortality and morbidity. Causes such as atherosclerotic foci, degeneration, and tissue diseases may play a role in the emergence of this condition [1]. The global pandemic caused by coronavirus disease 2019 (COVID-19) has been around for about a year and the disease still maintains its severity. Increased inflammation due to this virus can lead to various cardiovascular diseases such as cardiac damage, thrombosis, and cardiomyopathy [2].

Inflammation plays an important role in vascular plaque rupture. Therefore, it is known to play a role in the development of AAD [3]. In addition, a case of arteriopathy related to COVID-19 infection has been reported in a pediatric case [4]. In this case report, we present the successful surgical treatment of a patient with type 1 AAD in the early period after the COVID-19 infection.

## Case presentation

**Figure 1:**
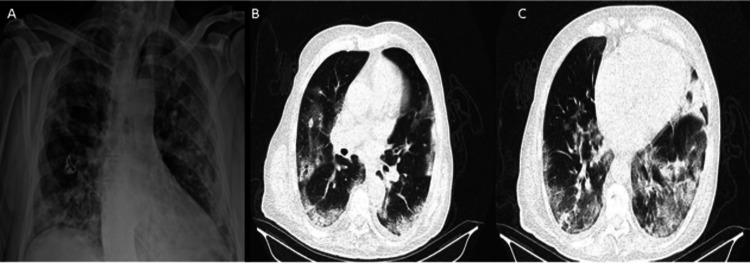
(A) Previous chest radiogram, (B, C) previous axial thorax tomography images of the patient with COVID-19 infection COVID-19: coronavirus disease 2019

A 62-year-old male patient was admitted to our emergency department with a complaint of chest pain radiating to his back. A dissection line starting from the ascending aorta was observed in the contrast-enhanced thoracoabdominal CT, and hence the patient was transferred to the intensive care unit (ICU). On physical examination, peripheral pulses of the lower and upper extremities were palpable. The arterial blood pressure measured in both arms was normal and similar (right arm: 170/90 mmHg; left arm: 175/90 mmHg). His past medical history was notable for chronic atrial fibrillation, diabetes mellitus, and lower extremity deep venous insufficiency. There was no history or sign of any connective tissue disease. The current medical treatment of the patient included insulin, calcium dobesilate, low molecular weight heparin, and beta-blocker. The patient had a history of hospitalization for seven days in the outpatient clinic and 10 days in the ICU due to COVID-19 infection with severe lung involvement (Figures 1A, 1B, 1C), with his polymerase chain reaction (PCR) being positive. The patient had been discharged at 20 days. Additionally, he had received steroid treatment (methylprednisolone 16 mg/day) due to ongoing respiratory symptoms.

**Figure 2:**
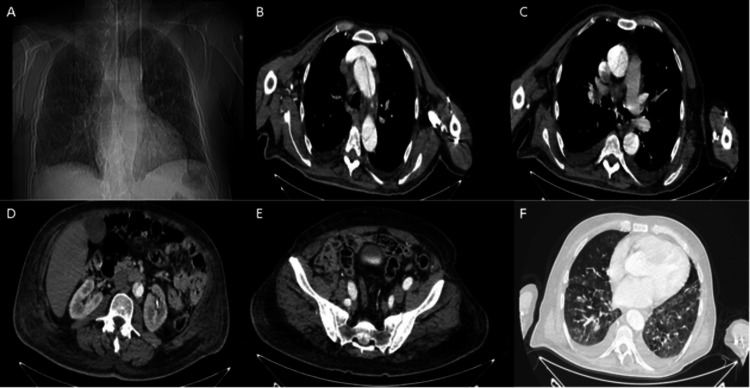
(A) Current chest radiogram, (B, C) current axial contrast-enhanced thoracoabdominal CT images of the patient, (D, E) a dissection line progressing to the iliac bifurcation, (F) axial thorax tomography images of the patient with sequela images of lung involvement areas due to COVID-19 infection CT: computed tomography; COVID-19: coronavirus disease 2019

He was evaluated with chest radiography (Figure 2A), transthoracic echocardiography, and thoracoabdominal CT tests. In contrast-enhanced thoracoabdominal CT, a dissection line starting from the ascending aorta and progressing to the iliac bifurcation was observed (Figures 2B, 2C, 2D, 2E). Also, sequela images of lung involvement areas due to COVID-19 infection were observed on CT images (Figure 2F). On transthoracic echocardiography, the ejection fraction was 60%, and a dissection flap in the ascending aorta was observed. The left ventricular end-diastolic diameter was 62 mm, the end-systolic diameter was 43 mm, and the left atrial diameter was 52 mm. No pathological finding was observed in the heart valves. Preoperative blood parameters were as follows: lactate dehydrogenase: 417 U/L; aspartate aminotransferase: 15; alanine aminotransferase: 10; albumin: 39.2 g/L; urea: 22 mg/dL; creatinine: 1.19 mg/dL; white blood cells: 11.26 10^3^/µL; neutrophils: 10.04 10^3^/µL; lymphocytes: 0.61 10^3^/µL; hematocrit: 40%; and platelets: 178 10^3^/µL.

**Figure 3:**
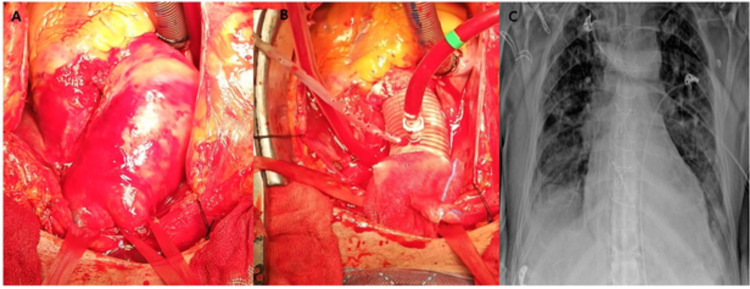
(A) Operative image of the dissected ascending aorta, (B) image of the aortic graft, (C) early postoperative chest radiogram image

A cardiopulmonary bypass with right axillary artery-venous two-stage cannulation and moderate hypothermia (28 °C) was performed on the patient emergently and under general anesthesia after he underwent a median sternotomy. Heparinization (300 IU/kg) produced an active clotting time of over 400 seconds. The ascending aorta was isolated (Figure 3A). A left atrial vent was placed. Cardiac arrest was achieved with cold blood cardioplegia (BC) (10-15 mL/kg, approximately 1,000 ml), given both antegrade and retrograde. Cardiac arrest was sustained with BC (300 ml), which was applied every 15-20 minutes. An aortotomy was performed and both the ascending aorta and transverse arch were replaced with a 30-mm polytetrafluoroethylene tube graft (Figure 3B). The distal anastomosis was performed under selective antegrade cerebral perfusion (20 minutes) with moderate hypothermia. The heart was successfully defibrillated. Cardiopulmonary bypass was weaned with low-dose inotrope support. Surgical incisions were closed by placing tube drains. The patient was admitted to the cardiovascular surgical ICU after the operation; a chest radiogram was obtained (Figure 3C), and the patient received standard postoperative care. Following the provision of hemodynamic stability, extubation was performed at the 36th hour postoperatively. He was discharged home 15 days postoperatively.

## Discussion

In this current case report, we shared the experience related to the surgical treatment of type 1 AAD in a patient who had contracted COVID-19 within a month beforehand but had only mild respiratory symptoms. The effects of this infectious disease, which has impacted the whole world for about a year, on the cardiovascular system are emerging day by day.

Inflammation plays an important role in the development of aortic dissection, as found in many patients. Although dissection may occur due to collagen tissue diseases, it is often caused by the rupture of an atherosclerotic plaque as the blood passes through the intima-media layers and progresses distally. Also, inflammation plays an important role in this plaque rupture [1]. Serum proinflammatory cytokines are elevated in coronavirus cases, resulting in lung damage and an increase in microthrombotic events [5]. Furthermore, neutrophil infiltration has been demonstrated in lung tissues in COVID-19 cases during autopsies [6].

Cardiovascular problems resulting from coronavirus infection encompass acute coronary syndrome (due to coronary occlusion, thrombosis), myocardial damage without coronary artery disease, arrhythmias, heart failure, pericardial effusion, and thromboembolic events [7]. In our literature review, we did not find a case report that covers the early period after COVID-19 infection in a patient during the ongoing pandemic. However, serious renal and cardiac damages are associated with severe COVID-19 infections, and they significantly increase the mortality rates [8].

He et al. have reported about four type 1 AAD cases admitted to their clinic in the initial phase of the pandemic. Although the authors did not have a definitive diagnosis in these cases, they observed suspicious inflammatory changes in the lung images. The operative team implemented the necessary preventive measures and successfully performed the operations [9]. However, in these cases, there is no information about the COVID-19 PCR positivity, and the patients were evaluated as potential COVID-19 cases only due to preoperative suspicion raised by the lung images. A similar case with these findings was reported by Akgul et al. [10]. Our case is differentiated from these cases with the finding of a positive PCR one month prior to the patient's current presentation and the observation of an aortic dissection during the recovery period.

## Conclusions

The ongoing COVID-19 epidemic represents a unique healthcare crisis in the history of mankind. Hence, it is currently not possible to fully predict the problems that may be caused by COVID-19. In our case, we think that the inflammatory process caused by COVID-19 may have been a triggering factor for the development of an aortic dissection. Further studies are required to obtain confirmation regarding this hypothesis.
